# Emvolio - A battery operated portable refrigerator preserves biochemical and haematological integrity of biological samples in preclinical studies

**DOI:** 10.12688/f1000research.109134.2

**Published:** 2023-01-19

**Authors:** Swastika Maity, Jaya Aakriti, Suman Manandhar, Sharad B Anchan, Ashlesh Bhat, Mayur U Shetty, Yogendra Nayak

**Affiliations:** 1 Department of Pharmacology, Manipal College of Pharmaceutical Sciences, Manipal Academy of Higher Education, Manipal, Karnataka, 576104, India; 2 Blackfrog Technologies Private Limited, Manipal, Karnataka, 576104, India

**Keywords:** Emvolio, portable refrigerator, biologicals, storage, transportation

## Abstract

**Background:** Emvolio is a non-medical device, indigenously developed portable refrigeration for maintaining the internal temperature 2–8˚C. The Indian Patent Office has granted patent for applications such as preservation and transport of medicines, vaccines, food, beverages, dairy etc. Further, use of Emvolio can be utilized in transport and store biologicals to preserve their biochemical and cellular integrity.  The objective of this study was to evaluate the biochemical and haematological integrity of biological samples such as rat blood, serum and liver.

**Methods:** The steady temperature was maintained inside the Emvolio, and it was compared to that of thermocol and polypropylene boxes aided with frozen gel packs. The blood and liver samples were isolated from Wistar rats and kept in Emvolio, thermocol and polypropylene boxes for 10 hrs, and the temperature was monitored. The blood parameters, namely red blood cells (RBC), white blood cells (WBC), platelets, haematocrit, haemoglobin, mean corpuscular volume (MCV), mean corpuscular haemoglobin concentration (MCHC) and red cell distribution width (RDW), serum parameters like alanine transaminase, alkaline phosphatase, total protein, albumin, creatine kinase, blood urea nitrogen and liver parameters like superoxide dismutase (SOD), glutathione (GSH), catalase were estimated and compared.

**Results: **Emvolio maintained a constant inner temperature range of 2–8˚C, whereas a significant temperature variation was seen in thermocol and polypropylene boxes. There was no significant deviation in the parameters tested when samples were kept in Emvolio for six hours compared to the zero hour readings. In contrast, there was a significant deviation among the parameters for the samples kept in thermocol and polypropylene boxes for six hours compared to zero hour parameters.

**Conclusions:** Emvolio maintained constant temperature and preserved the biological integrity of rat blood, serum and liver. Thus, Emvolio can be efficiently used as a biological sample carrier, especially in preclinical studies.

## Introduction

Collection, storage and transportation of biologicals has become the chief investment by industry, academia and public research organizations. ^1^ Due to the highly sensitive nature of the biologicals to environmental conditions like heat and moisture, samples such as blood, serum, saliva, tissue, and organ samples are utilized in the hospital and medical industry to diagnose and experiment for preclinical or clinical studies. ^2^^,^^3^ The international agency for research on cancer (IARC) describe the storage conditions for different biologicals. ^4^ The guidelines for the transportation and preservation of biological products are as per the National Institute of Health (NIH), International Society for Biological and Environmental Repositories (ISBER), ^5^ Center for Biologics Evaluation and Research (CBER), and Food and Drug Administration ( FDA). The biological sample collection, storage, transportation and preservation includes multidisciplinary collaboration among experts in drug discovery, engineering, and other health professionals. The stocks for the use of biologicals in the health care sector is rising every day, especially in pharmaceutical sectors. ^6^ This condition has made the health care system rely on technology to ensure the maintenance of temperature. Over the past ten years, dependence on technology has increased for cold chain management. ^7^ Especially during the COVID-19 pandemic, the importance of cold-chain management became more prominent. ^8^ Generally, 2–8°C temperature is maintained by freezing, whereas for some products freezing should not be done. As per WHO guidelines, biologicals must not be frozen and should be transported and stored at 36–46°F (2–8°C). Particular reference to vaccines, ensuring compliance with temperature in all stages of storage and transport is a must. Researchers are using thermocol based units, polypropylene units, or insulated plastic containers extensively. The thermocol box and polypropylene chillers are the cheapest alternatives that researchers and hospitals can use to transfer biological and blood samples. But due to lack of adequate temperature control, unavailability of information of the sample inside without opening the box itself results in loss of effectiveness in transportation. Hence, technological advancement is required for maintaining the cold chain requirement of biologicals at their optimum level.

**Figure 1.**
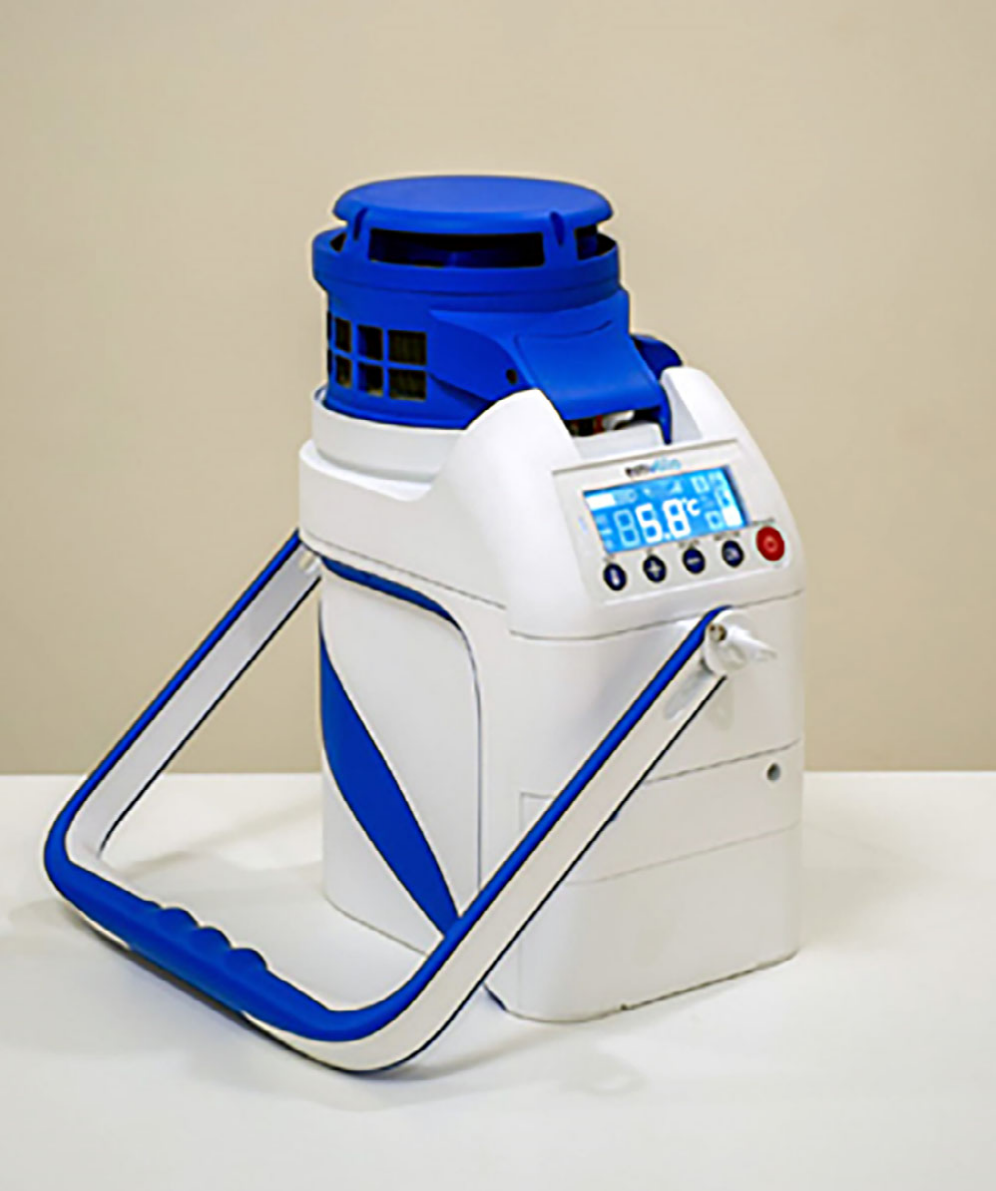
Emvolio portable refrigerator for transportation of biologicals/biological sample (product of Blackfrog technology Pvt Ltd, Manipal, India).

**Figure 2.**
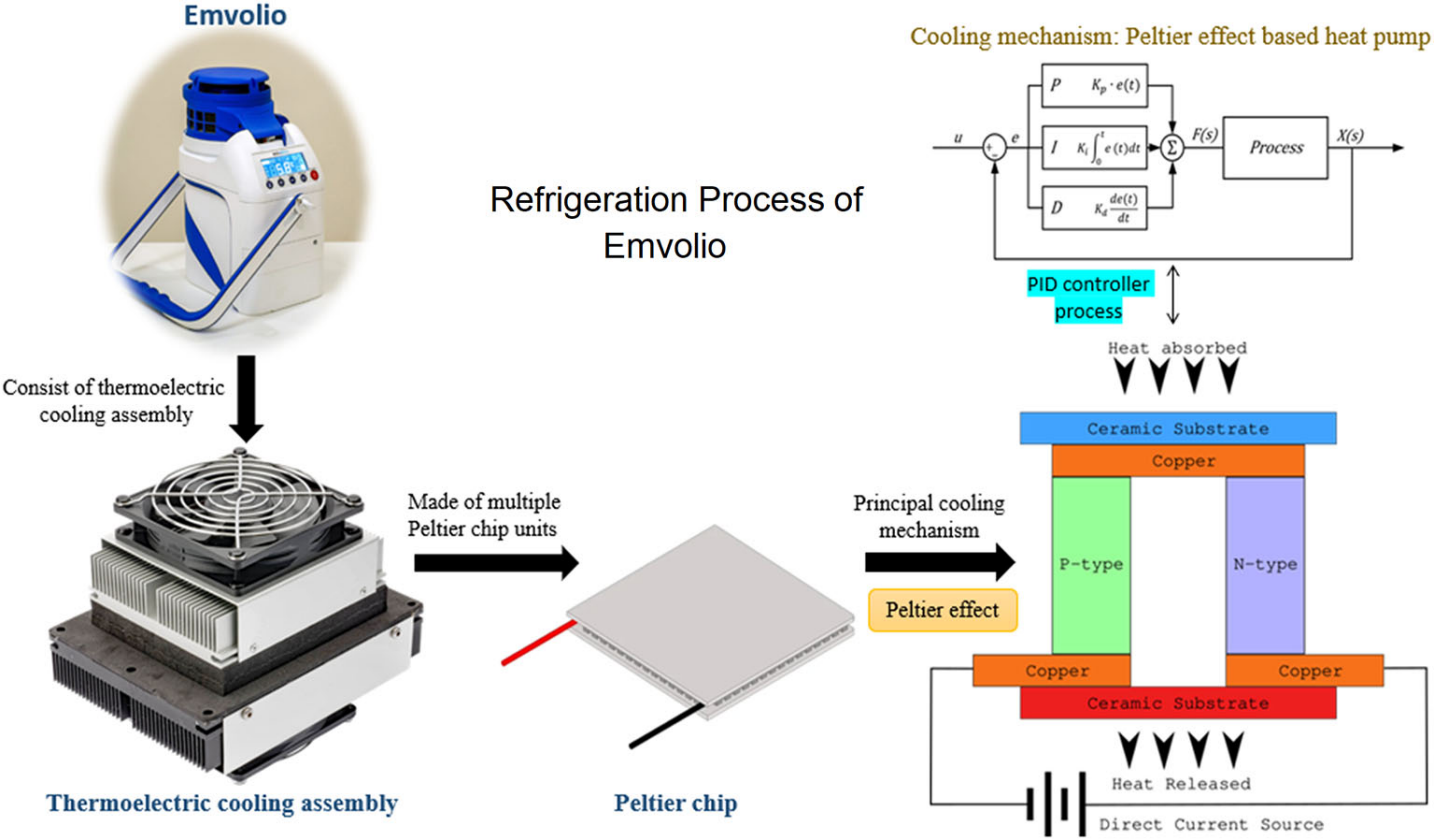
Thermodynamic mechanism used in Emvolio for its refrigeration technique of biological samples. The device has a cooling assembly made up of Peltier chips units. The cross-section of a simple Peltier effect based heat pump is presented in the figure. The blue side is the principal cooling mechanism known as the cold side, and the red as the hot side. Most Peltier chips have multiples of the structure in the figure in parallel to having higher power heat pumps. The device is linked with proportional–integral–derivative (PID) controller process to get accurate setpoint control and fast reaction to disturbance.

Emvolio is a portable active cooling device developed by Blackfrog technologies Pvt Ltd, Manipal, India, for the transportation, delivery and storage of biologicals, especially medicines, vaccines, food, beverages, dairy etc. However, this can also be used to preserve and transport serum, blood and culture samples that are required to be kept strictly between 2–8°C ( Figure 1). A 20 MAh rechargeable battery powers this portable device. This battery is roughly equivalent to a commercial cell phone battery. One of the primary requirements for the test is the autonomy of the battery for a minimum of 12 hours at extreme heat of 43°C ambient temperature. Emvolio was tested for the stringent WHO/PQS/E003/TS01.1 standard for a transportable, powered vaccine appliance at an independent WHO-accredited test facility. Further, the autonomy during temperature excursions due to human interference especially opening/closing the lid is also captured in the experiment. The reports for Emvolio was passed before it was tested for biological samples. Emvolio device gives 100% accountability to ensure temperature and aims to replace the standard thermocol-based carrier or polypropylene-based containers. This device can maintain 2–8°C for 10 hrs, have 1.5 L of capacity and weighs 5 kg. This technology supports rapid cooling and quick stabilization of temperature. It has 96% faster cooling capacity than ice-based technology in all weather conditions, and the device was tested by the company with operational capability at a temperature between –10°C to 43°C. Emvolio device works on a thermoelectric refrigeration principle and has an embedded temperature monitoring device controller, which helps in the dynamic temperature control feedback mechanism ( Figure 2). Emvolio has been certified by the Directorate General of Health Services, Ministry of Health and Family Welfare and National Regulatory Authority (NRA), India, following the Central Drugs Standard Control Organisation (CDSCO) guidelines as a non-medical device. The manufacturing company Blackfrog Technologies, Pvt. Ltd., also owns a patent for the same ( Indian patent no 201941013056).

The current objective was to estimate whether Emvolio can maintain steady temperature control of 2–8°C in comparison with thermocol, polypropylene-based boxes supported by ice packs and room temperature conditions. The objective was also to identify deviation in blood, serum and organ biochemical parameters stored in Emvolio, thermocol based boxes, polypropylene-based boxes supported by ice packs and room temperature.

## Methods

### Preparation of cooling devices

Emvolio was fully charged before the investigation. The gel pads were kept in a deep freezer for 24 hrs before the experiment. A temperature monitoring device, model RC-5 by Elitech, which follows the gold standard for monitoring thermal conductivity, was used. All four groups had their separate RC-5, Elitech devices for monitoring the temperature fluctuation. All three boxes were kept at room temperature. The RC-5, Elitech device was kept in Emvolio once it reached the temperature of 4°C as per the protocols set by the manufacturer. The RC-5, Elitech device was kept in the thermocol and polypropylene boxes 1 hr after keeping the gel pads as per the guidelines of cold chain management. The study was conducted forenoon 8.30–9 AM to 11.20–12 AM daily for seven days. Emvolio was charged after 9 hrs, and the gel pads were changed at 10 hrs. The data was collected each day after the experiment and analyzed.

Emvolio and the other alternatives were validated by the use of Q-tag ^®^. The Q-tag ^®^ is a range of WHO Performance Quality Safety (PQS) certified wireless temperature-sensors manufactured by Berlinger, a Swiss temperature monitoring solution company. Q-tag ^®^ is the gold standard for the cold chain monitoring system for biologicals and vaccines as per WHO.

### Procuring the rats and sample collection

**Institutional animal ethics committee (IAEC) approval**

All the applicable international, national and institutional guidelines for the care and use of animals were applied in the experiments. The experimental work was approved by IAEC (#IAEC/KMC/81/2019) of Manipal Academy of Higher Education (MAHE), and the experiments were carried out at the Central Animal Research Facility, Manipal, Karnataka, India (CPCSEA Reg no. 94/Po/ReBi/S/1999/CPCSEA). Rats were procured and acclimatized for seven days in 12 hrs light and dark cycle. Rats were fed with a standard pellet diet and water *ad libitum.* Each cage made up of polypropylene was used to cage two rats in each. The study was conducted as per the ARRIVE guidelines 2.0 using the ARRIVE Essential 10 checklist for pre-clinical animal studies ^9^ and all efforts were made to ameliorate any suffering of animals during the experiments.

**Experimental design**

Three months old female Wistar rats weighing 150–180 g were used for the study. The rats were sacrificed after anaesthetising with intraperitoneal ketamine and xylazine solution (9:1) solution. The blood was collected by cardiac puncture in small test tubes coated with anticoagulant 10% EDTA (Cat. No.: 10378-23-1; Sigma-Aldrich). EDTA 10% solution was made using Milli-Q water. Part of the blood was also collected in the Eppendorf tubes, and the serum was isolated after coagulation. The livers were perfused and isolated from the rat body. The samples of blood with anticoagulant, serum and liver were stored in a different container as per the following grouping. Each group contained n=6 female Wistar rats.

Group I: Emvolio (instrument for consideration)

Group II: Thermocol box padded with gel pads (common method for biological transfer in hospital and labs)

Group III: Polypropylene box with gel pads (common method for biological transfer in hospital and labs)

Group IV: Room temperature (control group)

**Figure 3.**
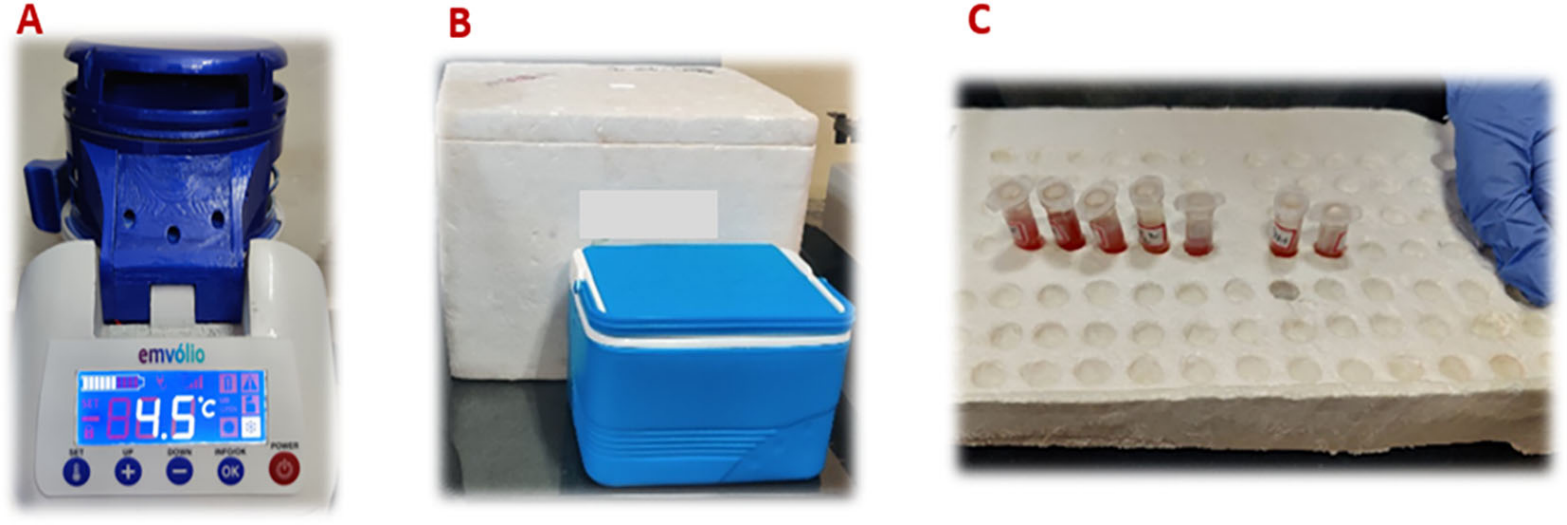
Containers of four groups, namely A: Emvolio; B: Thermocol box and Polypropylene box; C: Room temperature for the study loaded with biologicals.

The blood, serum and liver from each group of rats were stored in Emvolio, thermocol box, polypropylene box and room temperature. The storage at room temperature was kept as a control group for comparison ( Figure 3). The biochemical data from the groups thermocol box, polypropylene box, room temperature and Emvolio groups were compared.

**Evaluation of haematological parameters**

The RC-5, Elitech device was kept in Emvolio once it reached the temperature of 4°C as per the protocols set by the manufacturer Blackfrog technologies, Manipal. The RC-5, Elitech device was placed in the thermocol and polypropylene boxes to monitor the temperature fluctuations. Blood was collected in a test tube with 10% EDTA solution to avoid coagulation of blood ^10^ and was kept in the respective group containers. The blood samples were tested at a time interval of 3 hr intervals, 0 hr, 3 hr and 6 hrs, using the Veterinary blood cell counter (PCE-210VET, ERMA Inc., Tokyo, Japan). The blood samples were tested for total red blood cells (RBC), white blood cells (WBC), platelet, haemoglobin (Hb), haematocrit, mean corpuscular volume (MCV), mean corpuscular haemoglobin concentration (MCHC) and red cell distribution width (RDW). ^11^ The temperature of each box was noted after the biological sample analysis was completed.

**Evaluation of serum and organ parameters**

The serum and organs were preserved in their respective labelled containers for further investigation. The serum enzymes were analyzed using kits from Agappe, Sigma, and Elabsciences in the Agappe UV auto-analyser. The following kits were procured to conduct the study: blood urea nitrogen kit (Cat. No. 11610002), alanine transaminase (ALT) (Cat. No: 11409005; 11409006), alkaline phosphatase (ALP) (Cat. No: 11401010), lactate dehydrogenase (LDH) (Cat. No: 12011015), serum creatinine (creatinine) (Cat. No: 11420002), albumin (ALB) (Cat. No: 12011002) and total protein (TP). Around 1.5 mL of blood was collected for each sample of individual groups and was centrifuged at 8,000 rpm for 10 min to collect the serum sample. The parameters selected for the serum analysis were reported to be highly temperature-sensitive standardized studies; so, selective parameters were analyzed to find out the temperature variation effect on the serum sample of each group. ^12^

The liver sample was homogenized in 10% ice-cold KCl (150 mM) solution (Merck, Cat. No: 60142). ^13^ The homogenate was then centrifuged at 8,000 rpm for 10 min, and the supernatant was separated and kept in their respective labelled container (Emvolio, thermocol box, polypropylene box and room temperature). The samples for the group room temperature were kept on a centrifuge tube stand. Organ parameters were performed by homogenization of the liver sample and estivation of its antioxidant property. Superoxide dismutase (SOD), glutathione (GSH) analysis was done using the fluorescent plate reader (FLx800, Biotek), and catalase were estimated using UV spectrophotometer (UV-1650pc TBS instrument). This experiment was carried out throughout 12 hrs. Each sample for both serum and organ analysis was performed over a time interval of 3 hrs to estimate the effect of temperature on the biologicals. Therefore, the samples were analyzed at 0 hr, 3 hr and 6 hrs for their biochemical estimation.

### Statistical analysis

Statistical analysis was done using two-way ANOVA in GraphPad Prism version 8.0 p<0.05 was considered a significant difference for the comparison of all the parameters. The calculations which were done in GraphPad Prism can be done in Microsoft Excel by adding the free extension Analysis ToolPak by Microsoft ^®^ .

## Results

### Effect of temperature fluctuation during the storage

**Figure 4.**
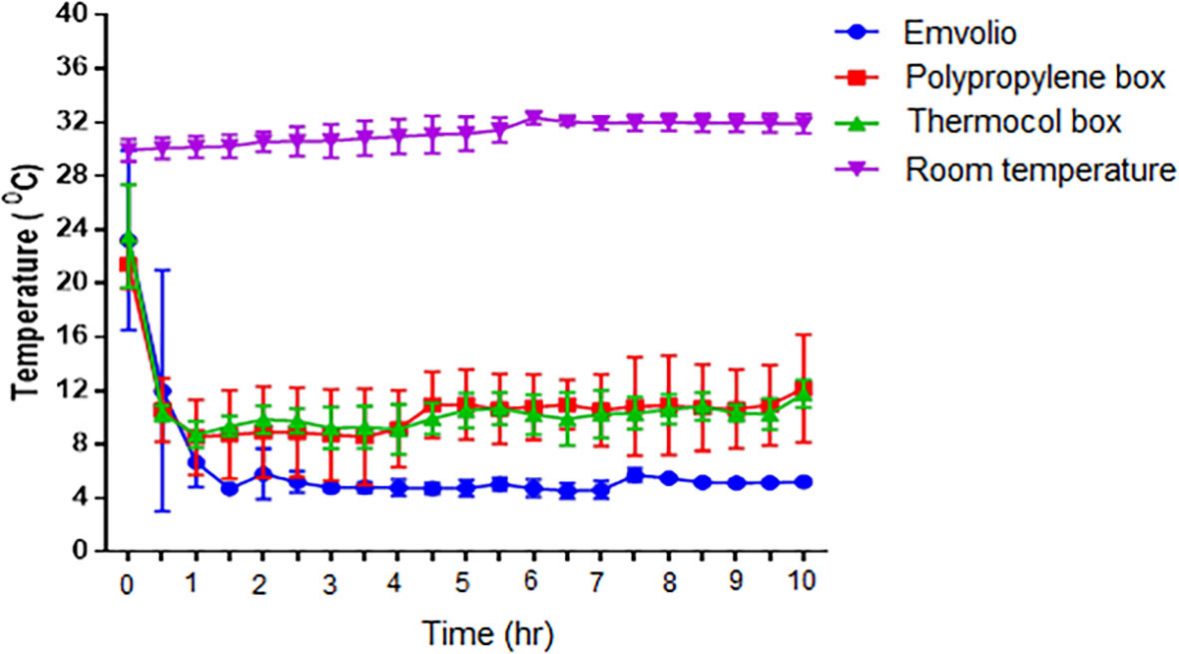
Graph of inner temperature vs time stating the different cooling systems, namely Emvolio, polypropylene box, thermocol box and room temperature.

The temperature recording done for 10 hrs over seven days in different cooling systems is represented in Figure 4. Constant maintenance of temperature within the range of 4–8°C was observed in the Emvolio cooling system kept at normal room temperature (30°C). Both gel-based cooling systems in the thermocol box and polypropylene box could not maintain the temperature in the 4–8°C range, and temperature fluctuations above 8°C was observed during the 10 hrs observation period. After the duration of the 4 hrs, in both polypropylene and thermocol boxes, an increase in the temperature from 8°C to 10°C was noticed. In contrast, the Emvolio cooling system maintained constant temperature (4–8°C) without significant deviations throughout the study duration.

### Effect of temperature on haematological parameters

Table 1 represents the changes observed in the blood cell concentrations analyzed in the blood samples stored at different storage systems. For the complete blood count analysis the blood parameters like RBC count, haemoglobin, haematocrit, WBC, MCV, MCHC, RDW were analyzed. ^9^ It was observed that at 6 hrs, RBC, haemoglobin and haematocrit values in the samples showed significant differences compared to 0 hrs. But WBC, MCV, MCHC and RDW did not produce any statistical changes compared to 6 hrs. The samples stored in the polypropylene box showed significant differences in the RBC and haemoglobin after 3 hrs and 6 hrs of storage. There was no significant change in WBC, MCV, MCHC and RDW during the observed duration. The samples stored in gel pad aided cooling in thermocol box showed a significant difference in the RBC after 3 hrs and 6 hrs of the sample storage ( Table 1). The samples stored at room temperature showed a significant increase in the RBC count, haematocrit after 3 hrs of storage, and a significant increase in RBC, haemoglobin, haematocrit, and MCV level 6 hrs after the storage. This signifies the effect of the storage temperature on the blood cell count.

**Table 1.** Effect of temperature fluctuations on the blood cells in the samples stored in different cooling systems.

	Emvolio			Polypropylene box			Thermocol box			Room temperature		
Analyte	0 hr	3 hr	6 hr	0 hr	3 hr	6 hr	0 hr	3 hr	6 hr	0 hr	3 hr	6 hr
RBC (10 ^6^/μL)	8.96±1.25	10.60±0.65 **	11.14±0.42 ***	9.23±0.38	10.80±1.29 **	12.06±0.93 ****	9.095±0.64	10.73±0.71 **	12.65±1.17 ****	9.56±0.35	11.35±0.91 **	13.93±0.77 ****
WBC (10 ^3^/μL)	3.60±1.85	6.28±1.248	4.27±1.607	5.7±1.69	8.25±2.94	8.43±2.577	6.15±3.09	6.87±2.32	6.62±3.531	7.60±3.41	10.05±5.78	9.067±4.37
Platelet (10 ^3^ μL)	626.67±93.25	567.67±219.87	475.17±166.34	369.33±165.02	485±178.29	585.33±179.01 *	566.5±142.07	482.833±167.25	434.67±183.04	504.17±157.24	512.17±198.83	460.00±216.74
Haemoglobin (g/L)	12.18±1.56	13.55±0.68	15.03±0.54 *	12.95±0.74	15.63±2.32 *	16.52±3.63 **	13.03±1.17	14.32±1.71	16.23±2.471 *	13.4±0.72	15.77±2.88	17.63±2.35 ***
Haematocrit (%)	47.43±1.85	55.32±3.06	57.57±3.549 *	48.03±4.24	52.75±6.06	58.27±9.92 *	45.28±3.26	52.94±4.26	56.5±9.422 **	47.58±2.96	61.42±4.45 ***	77.20±13.99 ****
MCV (fL)	50.95±2.36	51.32±1.83	51.37±1.89	50.10±1.87	50.87±2.04	52.933±3.688	49.73±1.44	50.17±1.64	50.05±6.456	49.68±1.55	52.15±1.51	59.00±2.78 ****
MCHC (%)	27.73±1.56	28.4±1.34	27.033	27.07±1.22	28.33±1.76	29.32±1.61	28.02±1.15	27.38±2.52	24.61±1.65	28.15±0.64	26.55±2.03	24.62±4.16 *
RDW (%)	15.63±0.42	16.03±0.50	16.25±0.67	15.92±0.40	16.42±0.40	16.38±0.73	16.00±0.14	16.53±0.51	16.52±0.64	16.12±0.50	16.25±1.31	16.38±0.73

Data are represented as Mean±SD, n=6.

* Represents p<0.05,

** p<0.01,

*** p<0.001,

**** p<0.0001 as compared with 0 hr concentrations.

### Effect of temperature on blood biochemistry

Among the estimated serum enzymatic parameters, only three parameters, namely alkaline phosphatase, total protein, and albumin, significantly varied with temperature increase. The serum samples stored at room temperature had increased alkaline phosphatase, total protein, and albumin after 6 hrs of collection compared to the 0 hr reading. However, no significant difference was observed in other estimated serum parameters during the observed duration. Serum alkaline phosphatase and total protein were significantly high in samples stored in thermocol box for 6 hrs. A significant change in the total protein was observed in the serum stored in thermocol box after 3 hrs. In contrast, other parameters had no significant changes during the storage condition. The serum stored in polypropylene box showed a significantly higher alkaline phosphatase and albumin after a 6 hr duration ( Table 2). The samples stored in Emvolio with well-maintained temperature showed fewer fluctuations in the serum parameters. However, a significant change in the alkaline phosphatase was observed and the device was able to maintain a stable enzyme concentration in the stored samples for about 6 hrs.

**Table 2.** Effect of temperature fluctuations on the blood serum parameters in the samples stored in different cooling systems.

Analytes	Emvolio			Polypropylene box			Thermocol box			Room temperature		
	0 hr	3 hr	6 hr	0 hr	3 hr	6 hr	0 hr	3 hr	6 hr	0 hr	3 hr	6 hr
Alanine transaminase, U/L	59.68±8.67	58.65±8.50	58.58±8.59	65.70±17.10	68.67±15.94	75.82±23.52	61.98±6.68	62.51±8.26	67.06±3.57	61.43±11.96	67.88±14.88	68.7±14.77
Alkaline phosphatase, U/L	3.23±0.14	3.31±0.15	3.40±0.13 *	3.39±0.27	3.46±0.23	3.60±0.17 **	3.19±0.575	3.32±0.44	3.45±0.35 ***	3.44±0.16	3.57±0.14	3.68±0.09 ***
Total protein, g/dL	6.62±0.23	6.63±0.23	6.63±0.15	6.18±0.15	6.45±0.25	6.33±0.92	6.08±0.22	6.68±0.20 *	6.81±0.64 **	6.35±0.26	6.53±0.33	6.97±0.48 **
Albumin, g/dL	3.22±0.06	3.17±0.08	3.10±0.08	3.13±0.26	3.10±0.25	3.33±0.29	3.41±0.128	3.34±0.10	3.37±0.133	3.40±0.267	3.34±0.22	3.63±0.17 *
Creatine kinase, U/L	0.30±0.04	0.30±0.05	0.32±0.023	0.28±0.098	0.31±0.131	0.28±0.12	0.29±0.06	0.35±0.09	0.31±0.094	0.27±0.06	0.27±0.06	0.29±0.08
Blood urea nitrogen, mg/dL	41.42±3.89	40.92±3.943	40.57±3.94	40.1±2.87	41.22±4.062	43.02±5.386	44.82±6.32	45.25±7.16	47.27±9.71	46.12±5.74	46.77±6.36	48.37±8.75

Data are represented as Mean±SD, n=6.

* Represents p<0.05,

** p<0.01,

*** p<0.001,

**** p<0.0001 as compared with 0 hr concentrations.

### Effect of temperature on tissue biochemistry

The liver samples stored in the different storage boxes were homogenized and used to estimate the markers like SOD, GSH and catalase. There were no significant differences in liver catalase and GSH when stored in Emvolio for 6 hrs. In contrast, SOD had a few fluctuations. The samples stored in polypropylene boxes showed a significant difference in SOD after 3 hrs and 6 hrs of storage. A significantly higher SOD was observed in the samples stored in the thermocol box after 3 hrs and 6 hrs, and GSH and catalase after 6 hrs of storage. Samples kept at room temperature showed a significant increase in the SOD level after 3 hrs and 6 hrs; catalase after 6 hrs of storage ( Table 3).

**Table 3.** Biochemical Estimation of organ parameters catalase, SOD and GSH stored in different cooling systems.

Analytes	Emvolio			Polypropylene box			Thermocol box			Room temperature		
	0 hr	3 hr	6 hr	0 hr	3 hr	6 hr	0 hr	3 hr	6 hr	0 hr	3 hr	6 hr
Catalase (IU/g Hb)	0.617±0.059	0.613±0.039	0.654±0.1	0.623±0.048	0.607±0.064	0.654±0.051	0.618±0.053	0.58±0.037	0.704±0.091 *	0.617±0.059	0.67±0.064	0.712±0.035 *
SOD (IU/g Hb)	0.473±0.033	0.529±0.074	0.636±0.081 ***	0.44±0.071	0.556±0.106 **	0.652±0.06 ****	0.468±0.028	0.575±0.08 *	0.587±0.091 **	0.5±0.043	0.737±0.102 ****	0.92±0.081 ****
GSH (μM/g Hb)	0.124±0.011	0.125±0.011	0.128±0.007	0.122±0.006	0.131±0.021	0.131±0.007	0.119±0.008	0.123±0.014	0.145±0.027 ****	0.119±0.006	0.117±0.006	0.126±0.007

Data are represented as Mean±SD, n=6.

* Represents p<0.05,

** p<0.01,

*** p<0.001,

**** p<0.0001 as compared with 0 hr concentrations.

## Discussion

Emvolio is a precision refrigeration system with an inbuilt internet-of-things (IoT) element that aids in remote-monitoring of vital statistics such as adherence to 2–8°C. The study conducted aimed to evaluate Emvolio for its efficacy as a biological carrier. The data was compared against the conventionally used non-medical biological carriers, such as thermocol boxes and polypropylene boxes filled with gel-pads. Room temperature was kept as the control group for our data analysis. The temperature recording done for a period of 10 hrs (over seven days) in different cooling systems is represented in Figure 4. Constant temperature within the range of 4–8°C was maintained only in the Emvolio cooling system compared to other systems. Both gel-based cooling system in the thermocol box and polypropylene box did not maintain the steady temperature in the 4–8°C range, and fluctuations above 8°C were observed during the 10 hrs period. A sharp increase in the temperature from 8°C to 10°C was noticed in the polypropylene box and thermocol box after the 4 hrs duration.

The serum samples stored at room temperature showed a significant increase in alkaline phosphatase (p<0.001), total protein (p<0.01), and albumin (p<0.05) 6 hr after the collection. A significant change in the total protein (p<0.05) level was observed in the serum stored in thermocol box after 3 hrs. In contrast, other parameters had no significant changes during the storage condition. The serum stored in the polypropylene box showed a significant increase in alkaline phosphatase (p<0.01) and albumin (p<0.05) after 6 hrs duration. The samples stored in Emvolio with well-maintained temperature showed fewer serum fluctuations and maintained stable enzyme concentration in the stored samples for 6 hrs. The organs stored in the different storage boxes were homogenized and the antioxidant markers, like SOD, GSH and catalase, were estimated. The samples stored in Emvolio showed a significant increase in SOD concentration (p<0.001) after 6 hrs of storage and no significant changes in the other parameters. The samples stored in polypropylene box showed a significant increase in SOD after 3 hrs (p<0.01) and 6 hrs (p<0.0001) of the storage. A significant increase in the SOD was observed in the samples stored in the thermocol box after 3 hrs (p<0.05) and 6 hrs (p<0.01), GSH (p<0.0001) after 6 hrs and catalase (p<0.05) after the 6 hr duration. Samples kept at room temperature showed a significant increase in the SOD level both after 3 hrs (p<0.0001) and 6 hrs (p<0.0001); and catalase (p<0.05) after 6 hrs of storage. Therefore, it can be inferred that Emvolio maintained a constant temperature of 2–8°C throughout the study with minimum fluctuation. Hence, it comes closest to meeting the requirements laid out by the Drug and Cosmetics Act 1940 for a biological carrier. Therefore, Emvolio is a better alternative to maintain the integrity of biological samples.

Gel pads are widely used in the manufacturing and health care industry as coolants. It helps in the maintenance of temperature below the freezing point of the stored sample. ^14^ The gel pad weighs approximately 500 g/pack and contains a liquid cocktail of chemicals that dampen heat conduction of the cold pack, allowing the gel pack to retain the heat, in return keeping its surroundings cool for a more extended period. As per cold chain management guidelines, it is recommended for the use of biologicals. The gel pads need to be frozen 24 hrs before use to act as an aid for the biological carrier. These gel pads are used in traditional transporters such as thermocol boxes and polypropylene boxes. The thermocol boxes are made up of Styrofoam. The heat transfer from the outside environment/surroundings makes the thermocol boxes an efficient heat insulator. Generally, a thermocol box is loaded with gel packs for the biological transportation technique. This helps retain the coolness for a more extended period. Styrofoam with an extremely low density of 890–910 kg/m, ^3^ when packed in between two plastic walls of polypropylene box, the cooling effect will be better. Further, polypropylene is a low density, chemical resistant, non-corrosive, high temperature resistant biodegradable material. Hence, polypropylene boxes are widely used in the healthcare industry to carry biologicals.

In a developing nation like India, with a high population and a majority residing in rural areas, biological samples are regularly transported over long distances for diagnosis. Research has assessed temperature during vaccine transfers at various levels under the Universal Immunization Program in 21 districts of India - Bihar, Kerala, and Gujarat. A significant episode of sub-zero exposure was reported, and the study concluded that rigorous monitoring of temperature control is essential to ensure vaccines potency and efficacy. ^15^ Thus, the thought of a technical solution for this is dependent on the availability of continuous electricity for the refrigeration unit to maintain the pre-set temperature and provide a conducive environment to biologicals with special reference to vaccines. However, poor connectivity and erratic power supply had rendered this unviable, especially in the context of rural India. ^16^

In Emvolio, the thermoelectric effect is the conversion of voltage difference to temperature difference and vice-versa. In a thermoelectric cooler, when a voltage difference is applied, a temperature difference is observed. The thermoelectric effect in it comprises three different outcomes: Peltier effect, Seebeck effect and Thomson effect. If a direct current is passed through the junction of two differently doped semiconductors, heat flux is generated, called a Peltier effect. This Peltier effect is used for the thermoelectric cooling and heating application. ^17^ Effectively this device is a heat pump moving heat from one side to the other when a current runs through it. The device has a thermoelectric cooler built around the Peltier chip to achieve cooling. In this system, two different heatsinks are attached to the hot side and the cold side to effectively release and absorb heat. To improve the heat exchange, on the heatsink, fans are mounted. Insulation foam is placed between the heatsinks around the Peltier chip to isolate the hot and cold heat sinks thermostatically. The main advantage of using a thermoelectric cooler over a compressor-based refrigerator is that it has no moving parts or circulating liquids. Further, the thermoelectric cooler is lightweight so makes a very compelling case for use in a portable system, ^18^ and a digital sensor in the cooling chamber is used for feedback. There are multiple ways to control the temperature of the chamber, Emvolio uses a method called proportional-integral-derivative (PID) controller. PID controller has been an industry standard due to its fast response, accurate setpoint control and fast reaction to disturbance. ^19^ In most cases, a traditional compressor-based refrigerator uses an on-off based controller, which creates an oscillation in the temperature, ^20^ which is not suitable for most biological samples. The PID controller consists of three different controllers in itself proportional, integral and differential. The proportional controller gives an output directly proportional to the error in the current temperature. ^21^ Due to limitations in the PID-controller a steady-state error in temperature may exist, which can be eliminated by an integral controller that sums up all the past errors to give a uniform output. The differential controller predicts the future behaviour of the temperature error and gives the output. Combining all these controllers into a PID controller and a digital sensor helps Emvolio maintain a strict temperature with an error of less than 0.5°C.

Blood, plasma, and serum analysis are key diagnostic activities in preclinical studies and are also followed in most hospitals to identify health problems. ^22^ Healthcare has drifted from high facility hospitals, to easy and affordable clinics, to ultimately home services with easy accessibility. Hence, it is now crucial that technology and medicine/healthcare goes hand-in-hand for better efficacy and minimizing the chances of error. ^23^ This could help transform an untenable health system into a tenable one. This will further reduce the chances of personal and environmental error and provide a faster and cheaper method for diagnosing biological samples and organ transportation. ^24^ To upgrade healthcare with technology, Emvolio can be an aid that can make the patient the point of care. It has the ability to support the diagnostics of one’s health at home and transport the sample to their physician at a far-off laboratory. Emvolio can not only fill the gap of the lack of technology access in the rural area, but also can help the healthcare workers to get accurate sample data with minimum damage. Pharma-logistics, as well as academia, have suffered from a lack of reliable last-mile transport. ^25^ So, technical support is required to aid the healthcare sector in coping with the storage and transportation facility of biologicals. Our study showed that Emvolio could maintain a constant temperature, and the samples stored in Emvolio showed significantly less deflection in the parameters estimated compared to thermocol box and polypropylene box aided with pre-frozen gel pads. The thermocol box and polypropylene box showed deviation from the required temperature range after 0 hrs, and thus, it was observed that the biochemical parameters showed fluctuation by the end of 6 hrs. Therefore, there is a critical need to upgrade the transportation and storage facility with appropriate technological support. Emvolio can be adapted for the safe transport of most thermally-sensitive biologicals, and it can be an ideal solution to upgrade the cold chain management system. Thielmann *et al.*, 2019 have carried out a study to verify the quality of vaccine refrigerator management, and they have followed the visual inspections of refrigerators used to store vaccines. As per their study, none of the practises fulfilled the quality criteria of standard practices. Important to them is the non-availability of temperature recording devices and logbooks. ^26^ Emvolio has inbuilt documentation and temperature recording system and can be used effectively.

Emvolio can be used for samples and specimen transportation for pandemic diseases like COVID-19 and minimize the risk of false negatives arising due to thermally-degraded samples or contamination. Emvolio head cap (lid) seals the device completely. The cold chamber isolates the inside environment from the outer world by using dual-layered stainless steel chambers that maintain a vacuum. So, Emvolio is a much safer option for the transportation of COVID-19 samples with minimal risk of exposure or cross-contamination. The inner metal walls of Emvolio makes it easy for the user to sanitize the device after use, and no additional technical training is required to handle the device. Emvolio is an ideal solution to maintain the integrity of the COVID-19 samples and shows promising potential for effective last-mile transport. The future smart healthcare networks are expected to combine the 5G and IoT devices and use drones from transportations. ^27^^,^^28^ We propose that Emvolio has the adaptability for these technologies.

**Figure 5.**
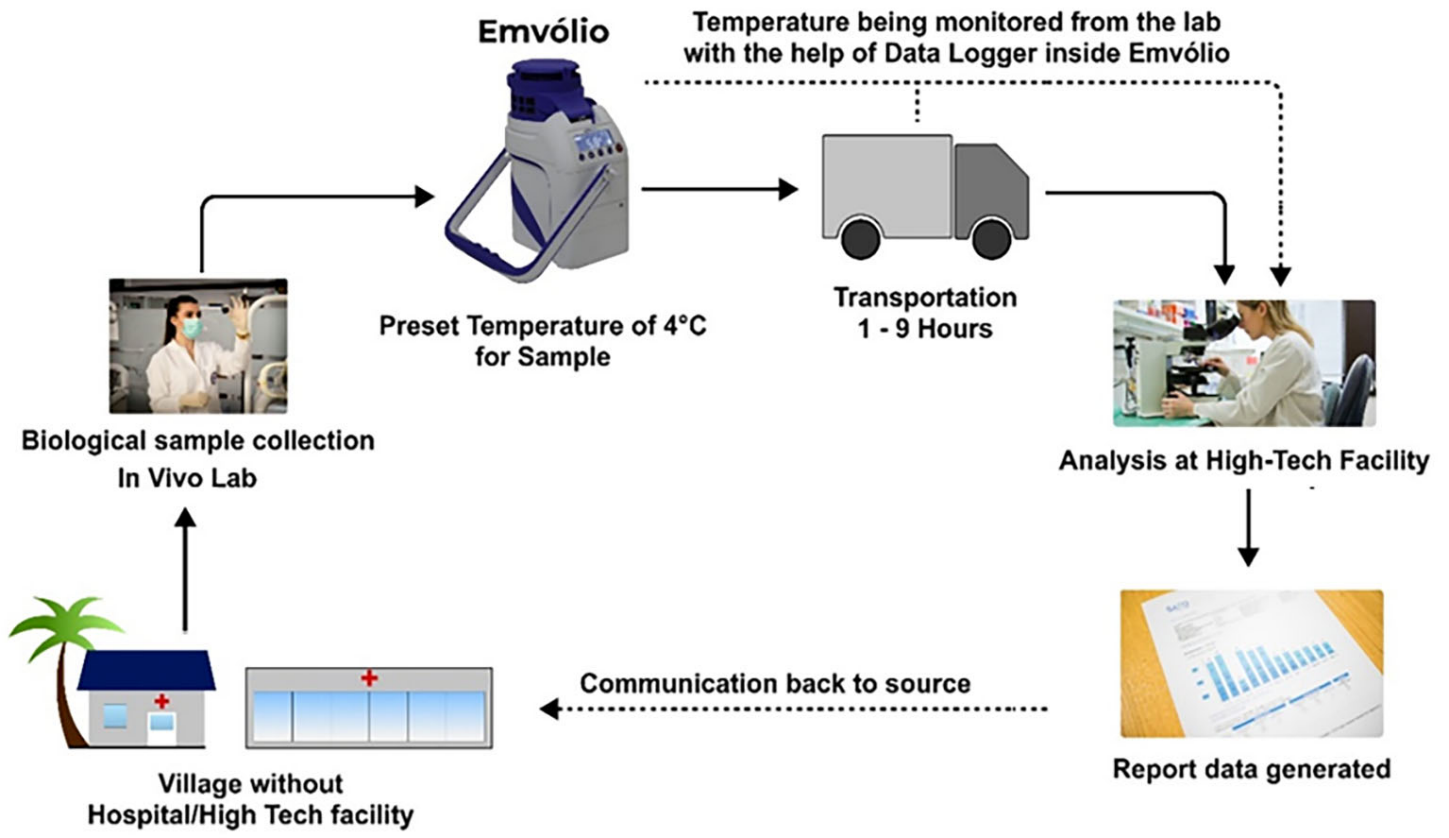
Proposed cold chain management of biologicals with Emvolio. The villages or remote labs do not have direct access to analysis facilities; Emvolio can serve as a supporting aid to transport the sample by maintaining the desired temperature. Such technology will reduce the chances of degradation of the sample and have a higher accuracy rate.

There was popular news that the Moderna vaccine requires -20°C and the Pfizer vaccine requires an even colder -80°C, it could be incompatible with typical vaccine carriers. However, these ultra-cold temperatures are meant for long-term storage and transport of the vaccines in the early parts of the supply chain. Incidentally, the Moderna vaccine is thermostable at 2–8°C up to 30 days, and the Pfizer vaccine is thermostable at 2–8°C up to 7 days as per the regulator’s data. This implies that regardless of their strict storage requirements, the last-mile transport will always be dependent on a 2–8°C cold chain, which Emvolio fulfils. Any vaccine, regardless of extreme storage temperatures, must be thawed before being administered to a recipient to avoid shock/injury. Once thawed, the vaccines cannot be refrozen again, which underlines the need for precision temperature control that other ice-based coolers fall short in. Thus, we propose cold chain management of biologicals with Emvolio, as depicted in Figure 5.

## Conclusion

Emvolio maintains the constant internal temperature between 2–8°C, preserving rat blood, serum, and liver for six hours and maintaining biological integrity. Thus, Emvolio can be efficiently used as a biological sample carrier, especially in preclinical studies.

## Data Availability

Mendeley Data: ‘Effect of temperature on hematological and biochemical parameters when the biological sample from rats kept in room temperature, thermocol, polypropylene box and innovative portable refrigerator’ https://doi.org/10.17632/vgdm3299fp.2. ^9^ This project contains the following underlying data:
• Temperature effect – Comparison of Biochemical & Hematological parameter_v2.xlsx (Effect of temperature fluctuations on the haematological parameters such as total RBC, WBC, platelets, and haemoglobin, haematocrit, MCV, MCHC, RDW in the blood samples, serum parameters such as ALT, ALP, total protein, albumin, creatinine kinase, blood urea nitrogen, and biochemical parameters in liver such as catalase, SOD and GSH stored in different cooling systems). Data are available under the terms of the Creative Commons Attribution 4.0 International license (CC-BY 4.0). Mendeley data: ARRIVE checklist for ‘Emvolio - A battery operated portable refrigerator preserves biochemical and haematological integrity of biological samples in preclinical studies’ https://doi.org/10.17632/vgdm3299fp. ^9^ Data are available under the terms of the Creative Commons Attribution 4.0 International license (CC-BY 4.0).
