# Supplementary material for: Emvolio - A battery operated portable refrigerator preserves biochemical and haematological integrity of biological samples in preclinical studies
Source: F1000Res. 2023 Sep 22;11:223. Originally published 2022 Feb 24. [Version 3] doi: 10.12688/f1000research.109134.3 (PMC10523100; doi:10.12688/f1000research.109134.3)
Supplement: Peer review history

# Reviewer report: Reviewer response for version 3

**DOI:** 10.5256/f1000research.156293.r208721

**Published:** 2023-09-26

**Authors:** Yuanheng Zhao

**Affiliations:** 1 The University of Tokyo, Bunkyo, Tokyo, Japan

The paper can be indexed.

Is the work clearly and accurately presented and does it cite the current literature?

Yes

If applicable, is the statistical analysis and its interpretation appropriate?

Partly

Are all the source data underlying the results available to ensure full reproducibility?

Yes

Is the study design appropriate and is the work technically sound?

Partly

Are the conclusions drawn adequately supported by the results?

Yes

Are sufficient details of methods and analysis provided to allow replication by others?

Partly

Reviewer Expertise:

Cryobiology

I confirm that I have read this submission and believe that I have an appropriate level of expertise to confirm that it is of an acceptable scientific standard.

# Reviewer report: Reviewer response for version 2

**DOI:** 10.5256/f1000research.143115.r173101

**Published:** 2023-07-14

**Authors:** Yuanheng Zhao

**Affiliations:** 1 The University of Tokyo, Bunkyo, Tokyo, Japan

Cold storage and transportation of biologicals are important in the field of biomedical engineering. Emvolio is a non-medical refrigeration for preserving samples by maintaining the internal temperature at 2–8˚C. This research paper titled “Emvolio - A battery operated portable refrigerator preserves biochemical and haematological integrity of biological samples in preclinical studies”, evaluated the biochemical and haematological integrity of biological samples such as rat blood, serum and liver after cold storage using inside the refrigerator.

 I think this paper can be considered to be accepted after revision. The authors did many experiments and the results are original. However, the significance and impact are moderate, especially the preservation procedures and the storage box used in the study is very normal and the result obtained is common sense.

 The biggest problem is that the article lacks innovation and it seems like an advertisement for a business products of a company instead of an academic paper.

 Some major flaws require immediate attention:

**1) Abstract- Background**

 “ *Emvolio is a non-medical device, indigenously developed portable refrigeration for maintaining the internal temperature 2–8˚C*”.

 It is better to delete the word “indigenously developed”. The cold storage box is not a novel concept. There are many business companies around the world that supply this kind of product for preservation at 4C.

**2) Introduction**

 “ *Generally, 2–8°C temperature is maintained by freezing, whereas for some products freezing should not be done.*”

 Replace the term “freezing” with “cooling”. “Freezing” means reducing temperature below 0C. Here, 2-8C is the unfrozen state.

**3)** I suggest the authors delete this paragraph, from “ *Emvolio is a portable active cooling device developed by Blackfrog technologies Pvt Ltd, Manipal, India, for the transportation, delivery and storage of biologicals, especially medicines, vaccines, food, beverages, dairy etc. …………….. The manufacturing company Blackfrog Technologies, Pvt. Ltd., also owns a patent for the same (Indian patent no 201941013056)*”. This is a research paper, which will be published for the academy exchange instead of an advertisement platform for a business company. Therefore, please delete all the products introduction or recommendation slogans for this company.

 The authors can review the study progress of cold storage boxes from the literature. Point out the disadvantages of the current business device, which can be included as one new paragraph and put here.

**4) Figure 4. Graph of inner temperature vs time.**

 Please state which kind of thermometer was used and where to put it inside the box. The specific position of the thermometer probe should be stated. And how many times of experiment was repeated.

**5) Statistical analysis and Table 1, 2, 3.**

 And how many times of experiment was performed? Which data was selected here, the best? Average value?

 Above all, I strongly suggest the authors reduced the content of advertisements for the business products of Emvolio. Put more focus on the viability parameter of samples during preservation.

 Finally, the title can be “Assessment of biochemical and haematological integrity of biological samples (in preclinical studies) after preservation using a battery operated portable refrigerator”

Is the work clearly and accurately presented and does it cite the current literature?

Yes

If applicable, is the statistical analysis and its interpretation appropriate?

Partly

Are all the source data underlying the results available to ensure full reproducibility?

Yes

Is the study design appropriate and is the work technically sound?

Partly

Are the conclusions drawn adequately supported by the results?

Yes

Are sufficient details of methods and analysis provided to allow replication by others?

Partly

Reviewer Expertise:

cryobiology

I confirm that I have read this submission and believe that I have an appropriate level of expertise to confirm that it is of an acceptable scientific standard, however I have significant reservations, as outlined above.

# Reviewer report: Reviewer response for version 2

**DOI:** 10.5256/f1000research.143115.r160833

**Published:** 2023-01-23

**Authors:** Ciro Aprea

**Affiliations:** 1 Department of Industrial Engineering, University of Salerno, Salerno, Italy

The authors have answered to my questions correctly. The paper is now acceptable for indexing, in my opinion.

Is the work clearly and accurately presented and does it cite the current literature?

Yes

If applicable, is the statistical analysis and its interpretation appropriate?

Not applicable

Are all the source data underlying the results available to ensure full reproducibility?

Yes

Is the study design appropriate and is the work technically sound?

No

Are the conclusions drawn adequately supported by the results?

No

Are sufficient details of methods and analysis provided to allow replication by others?

Yes

Reviewer Expertise:

Applied thermodynamics, Refrigeration

I confirm that I have read this submission and believe that I have an appropriate level of expertise to confirm that it is of an acceptable scientific standard.

# Reviewer report: Reviewer response for version 1

**DOI:** 10.5256/f1000research.120598.r150286

**Published:** 2022-10-06

**Authors:** Ciro Aprea

**Affiliations:** 1 Department of Industrial Engineering, University of Salerno, Salerno, Italy

The title of the paper is "A battery operated portable refrigerator preserves biochemical and haematological integrity of biological samples in preclinical studies". In this paper the authors present a refrigerated system equipped with a Peltier module finalized to vaccine preservation in the range 2°C-8°C. The aim of their study is to obtain a system capable of maintaining the vaccine storage temperature when the system is picked up from a refrigerated space where it has been located to reach the correct storage temperature. The Peltier module can contribute to maintain the "cold chain".

 The device presented is interesting but some aspects have to be evidenced. 

1. Which is the autonomy of the battery of the refrigerator when the ambient temperature is of 32°C?
2. And the autonomy of the battery when the refrigerator is opened and closed every hour for thirty seconds (simulation for withdraw the vaccine inside)
3. In this last case which is the time necessary for restore the correct temperature?
4. In this last case which is the maximum temperature reached with reference to the temperature set-point?
5. The temperature of 2°C-8°C are useful only for some vaccines. For Covid-19 the correct conservation temperature of some vaccines are also -20°C (Moderna for example).

Is the work clearly and accurately presented and does it cite the current literature?

Yes

If applicable, is the statistical analysis and its interpretation appropriate?

Not applicable

Are all the source data underlying the results available to ensure full reproducibility?

Yes

Is the study design appropriate and is the work technically sound?

No

Are the conclusions drawn adequately supported by the results?

No

Are sufficient details of methods and analysis provided to allow replication by others?

Yes

Reviewer Expertise:

Applied thermodynamics, Refrigeration

I confirm that I have read this submission and believe that I have an appropriate level of expertise to confirm that it is of an acceptable scientific standard, however I have significant reservations, as outlined above.

# Response

**Published:** 2022-10-26

**Authors:** Yogendra Nayak

**Affiliations:** Manipal Academy of Higher Education, India

Dear Ciro Aprea,

 We thank for your valuable questions and suggestions. Authors are obliged to the reviewer comments. Followings are the answers to your comments.

**Q1.** Which is the autonomy of the battery of the refrigerator when the ambient temperature is of 32°C?

**Response:** The product (Emvolio) has been tested and successfully passed the stringent WHO/PQS/E003/TS01.1 standard for a transportable, powered vaccine appliance. One of the primary requirements for the test is the autonomy of the battery for a minimum of 12 hours at extreme heat of 43°C ambient temperature. Please see the archived test reports at an independent WHO-accredited test facility wherein Emvolio ran for 13+ hours.

**Q2.** And the autonomy of the battery when the refrigerator is opened and closed every hour for thirty seconds (simulation for withdraw the vaccine inside)

**Response: **The autonomy during temperature excursions due to human interference is also captured in the above-mentioned report.

**Q3.** In this last case which is the time necessary for restore the correct temperature?

**Response: **We have conducted internal testing to determine the rate of temperature excursions and cool-down period due to human interference, i.e. opening/closing the lid. The results have also been contrasted against routinely used ice-based refrigeration systems. This report is also archived in a similar manner.

**Q4.** In this last case which is the maximum temperature reached with reference to the temperature set-point?

**Response: **The maximum temperature reached typically is dependent on the ambient temperature. We conducted our interference testing at 34°C, wherein the maximum temperature inside the cold chamber was between 9-11°C.

**Q5.** The temperature of 2°C-8°C are useful only for some vaccines. For Covid-19 the correct conservation temperature of some vaccines are also -20°C (Moderna for example).

**Response: **There is a popular misconception that since the Moderna vaccine requires -20°C and the Pfizer vaccine requires an even colder -80°C, it could be incompatible with typical vaccine carriers. These ultra-cold temperatures are meant for long-term storage and transport of the vaccines in the early parts of the supply chain. Incidentally, the Moderna vaccine is thermostable at 2-8°C up to 30 days, and the Pfizer vaccine is thermostable at 2-8°C up to 7 days as per the regulator’s data (For reference, click here for the report). This implies that regardless of their strict storage requirements, the last-mile transport will always be dependent on a 2-8°C cold chain, which Emvolio fulfils. Any vaccine, regardless of extreme storage temperatures, must be thawed before being administered to a recipient to avoid shock/injury. Once thawed, the vaccines cannot be refrozen again, which underlines the need for precision temperature control that other ice-based coolers fall short in.

 Further, we thank the reviewer for the input which made the reader a clear idea about the paper.

 Authors are obliged to the reviewer comments
